# Validation of the diagnostic accuracy of a urine‐based DNA methylation marker test in patients with upper urinary tract lesions

**DOI:** 10.1002/bco2.70195

**Published:** 2026-03-19

**Authors:** Christian Daniel Fankhauser, Kilian Röthlin, Philipp Baumeister, Agostino Mattei, Paolo Piatti, Yap Ching Chew, Ernest Kaufmann

**Affiliations:** ^1^ Faculty of Health Sciences and Medicine University of Lucerne Lucerne Switzerland; ^2^ Clinic for Urology University Teaching and Research Hospital of the University of Lucerne Lucerne Switzerland; ^3^ University of Zurich Zurich Switzerland; ^4^ Clinic for Urology Kantonsspital Winterthur Winterthur Switzerland; ^5^ Zymo Research Corp Irvine California USA; ^6^ Pangea Laboratory LLC Tustin California USA

**Keywords:** DNA methylation, non‐invasive testing, upper tract urothelial carcinoma, urine marker, UTUC

## Abstract

**Objectives:**

This study aims to validate the diagnostic accuracy of a novel urine‐based DNA methylation test in patients with suspected upper tract urothelial carcinoma (UTUC) on CT urography and to assess its potential to eliminate the need for diagnostic ureterorenoscopy (URS) in selected patients, expedite treatment and identify high‐grade tumours suitable for neoadjuvant chemotherapy.

**Patients and Methods:**

We prospectively collected urine samples from 46 consecutive patients with suspected UTUC in computed tomography and analysed them using the Bladder CARE™ methylation test. Test performance was evaluated against final pathology from URS biopsies and/or surgical specimens. We performed Youden Index analysis to optimise diagnostic cut‐off values and assessed correlations between Bladder CARE Index (BCI) levels and tumour characteristics, particularly grade differentiation.

**Results:**

Using the manufacturer's cut‐off (BCI > 2.5), the test demonstrated 95% sensitivity, 69% specificity, 70% positive predictive value and 95% negative predictive value (NPV), significantly outperforming cytology (11% sensitivity). An optimised, study‐derived cut‐off (4.35) further improved specificity to 92% with sensitivity and NPV remaining ≥95%. Importantly, a higher threshold (BCI > 10) yielded 100% specificity and 100% PPV, although at the expense of sensitivity (65%). Median BCI values differed between high‐grade (38.6) and low‐grade tumours (9.45), suggesting utility for non‐invasive grade assessment. BCI also correlated with tumour size (β = 12 mm per log10 increase, *p* = 0.08).

**Conclusion:**

This novel urine‐based DNA methylation test offers high diagnostic accuracy for UTUC detection. However, clinical interpretation should be threshold dependent. While BCI values >2.5 show high sensitivity, the PPV of 70% indicates a relevant proportion of false‐positive results, and diagnostic URS remains warranted in this range. In contrast, high positive values (BCI > 10) demonstrated 100% specificity and PPV and could enable direct progression to definitive surgery without diagnostic URS, avoiding procedure‐related complications and expediting treatment. The correlation with tumour grade addresses a critical need for identifying candidates for neoadjuvant chemotherapy without invasive tissue diagnosis.

## 1 INTRODUCTION

Upper tract urothelial carcinoma (UTUC) poses distinctive diagnostic challenges that frequently require multiple diagnostic evaluations, often culminating in invasive procedures.^1^ Ureterorenoscopy (URS) with biopsy remains the diagnostic reference standard but is associated with considerable morbidity, including ureteric injury, infection and haematuria, as well as potential tumour seeding and intravesical recurrence.^2^, ^3^, ^4^ In addition, repeated or prolonged diagnostic workups may delay definitive treatment, increasing the risk of both over‐ and under‐treatment. These limitations highlight the need for accurate, non‐invasive diagnostic tests that can reduce morbidity and minimise treatment delays.^1^ In this context, Bladder CARE™, a urine‐based methylation test for urothelial cancers, has emerged as a promising diagnostic tool. Previous studies have reported high diagnostic accuracy for UTUC, with sensitivity of 96%, specificity of 88%, positive predictive value (PPV) of 89% and negative predictive value (NPV) of 96%, leading to FDA Breakthrough Device Designation in December 2023.^5^, ^6^ This study aims to validate the diagnostic accuracy of this methylation‐based urine test in a European patient cohort.

## 2 PATIENTS AND METHODS

We conducted prospective sample collection with retrospective analysis of all consecutive patients with suspected UTUC based on computed tomography urography (CTU) imaging between December 2023 and August 2025. All included patients had preoperative Bladder CARE™ test results available. Although results were reported to treating clinicians, they did not influence clinical decision‐making. Based on clinical circumstances, patients subsequently underwent diagnostic URS and/or definitive therapy.

Urine specimens were collected using standardised collection tubes containing preservatives provided in the Bladder CARE Urine Collection Kit (Pangea Laboratory, Tustin, CA) and shipped at ambient temperature via standard mail to Pangea Laboratory for analysis. Urine was obtained by bladder voiding during consultation or bladder catheterisation immediately before diagnostic URS. The Bladder CARE™ assay quantifies methylation levels of three urothelial cancer DNA biomarkers (TRNA‐Cys, SIM2 and NX1‐1) and generates a Bladder CARE™ Index (BCI), reported as a continuous numerical value. Results were classified into three diagnostic categories: negative (BCI < 2.5), low positive (BCI 2.5–10.0) and positive (BCI > 10.0). Low positive results indicate an increased likelihood of malignancy compared with negative cases, although they exhibit lower disease specificity and clinical severity than positive results with a BCI >10. The inclusion of both low positive and positive categories allows for differentiation of biological signal strength and clinical relevance. For performance evaluation, low positive and positive results were combined and classified as test positive to enable binary performance assessment. Urinary cytology was evaluated using the Paris System for Reporting Urinary Cytology.^7^ For analysis purposes, specimens reported as ‘Negative for High Grade Urothelial Carcinoma’ (NHGUC) or ‘Atypical Urothelial Cells’ (AUC) were classified as negative, while those reported as ‘Suspicious for High Grade Urothelial Carcinoma’ (SHGUC) or ‘High Grade Urothelial Carcinoma’ (HGUC) were classified as positive.

Exclusion criteria included: (i) prior radical cystectomy with urinary diversion using bowel segments; (ii) recurrent bladder cancer, especially recurrent carcinoma in situ (CIS), to avoid false‐positive results in inconspicuous cystoscopies; and (iii) patients unwilling or unable to undergo further diagnostic procedures due to medical reasons.

This study was conducted in accordance with the ethical principles outlined in the Declaration of Helsinki and approved by the institutional review board of the Swiss Ethical Committee (BASEC‐ID: 2025‐01778). All participants provided written informed consent.

### 2.1 Sample size and power considerations

An a priori power analysis was performed for sensitivity and specificity using exact one‐sided binomial tests (α = 0.05). Expected test performance was based on previously published Bladder CARE™ validation studies, reporting sensitivities of approximately 96% and specificities of approximately 88%.^6^ Clinically relevant minimum performance thresholds were defined with reference to standard urinary cytology, which achieves a sensitivity of approximately up to 70% for UTUC detection.^8^ Given the intended role of the test as a non‐invasive tool to reliably identify UTUC and potentially avoid diagnostic URS, sample size planning prioritised sensitivity over specificity. The study was therefore powered to demonstrate sensitivity above a minimum threshold of 0.70 and specificity above 0.65. Under these assumptions, at least 19 UTUC cases were required to achieve ≥90% power for sensitivity and at least 24 non‐UTUC cases to achieve ≥80% power for specificity. The anticipated sample size was driven by feasibility and consecutive recruitment of patients with suspicious CT urography findings at a tertiary referral centre. As patients with suspicious imaging but ultimately no UTUC are inherently limited, specificity was weighted secondary to sensitivity in the power considerations.

### 2.2 Statistical analyses

Demographic data for continuous variables were analysed using the Shapiro–Wilk test to assess normal distribution. Sensitivity, specificity, positive predictive value (PPV) and negative predictive value (NPV) of cytology and the Bladder CARE™ test were calculated based on true‐positive, true‐negative, false‐positive and false‐negative cases. The Youden Index was calculated to identify an alternative optimal cut‐off value. A receiver operating characteristic (ROC) curve was generated and area under the curve (AUC) was calculated for alternative BCI cut‐off values. To evaluate correlation between BCI and tumour size, linear regression analysis was performed. Due to anticipated skewed distribution of BCI values with large fluctuations, logarithmic transformation (log10) was applied to normalise the data before regression analysis. Tumour size was determined as follows: For surgically removed tumours (e.g., nephroureterectomy or ureterectomy), the largest diameter from the pathology report was utilised; when unavailable, the largest diameter from CT imaging was used. Additionally, a Wilcoxon rank‐sum test was performed to assess association between BCI and tumour grading from pathology specimens, classified as low‐grade or high‐grade. Statistical analyses were performed using R Studio (R Core Team, 2022, Version 2023.03.0 + 386).

## 3 RESULTS

Between December 2023 and August 2025, we collected urine samples from 46 patients with suspected UTUC on CTU (Table 1). The cohort had a mean age of 70 years (SD: 11) and mean Charlson Comorbidity Index of 5 (SD: 2), with 33% female patients and 26% current smokers. Presenting symptoms included visible haematuria (*n* = 17), non‐visible haematuria (*n* = 24) and pain/incidental findings on imaging (*n* = 26). UTUC was excluded by diagnostic URS in 16 patients, while 15 had URS findings suggestive of cancer. Ten patients were considered negative without URS after repeated imaging showed resolved lesions. Five patients did not undergo URS as imaging showed large tumours without any potential for kidney‐preserving surgery, and they were scheduled directly for definitive treatment. Treatment consisted of radical nephroureterectomy in 17 patients, partial ureterectomy in two patients, nephrectomy in four patients and transurethral resection of a distal tumour bulging out of the ureteral orifice in one patient.

**TABLE 1:** Baseline characteristics of the study population.

Variables	*N* = 46
Gender	
Female	15 (33)
Age	70 (SD: 11)
Ethnicity	
Caucasian	46 (100)
Medical history	
Charlson Comorbidity Index CCI	5 (SD: 2)
Visible haematuria	17 (37)
Non‐visible haematuria	24 (52)
Pain or incidental finding	26 (57)
Current smoking	12 (26)
Location cytology	
No cytology	7 (15)
Cytology left upper tract	8 (17)
Cytology right upper tract	13 (28)
Voiding cytology	4 (9)
Cytology catheterisation	14 (30)
Result urinary cytology (*n* = 41)	
Negative for high grade urothelial carcinoma (UC)	36 (78)
Atypical urothelial cells (AUC)	3 (7)
Suspicious for high grade UC	1 (2)
High grade UC	1 (2)
Location tumour	
Ureter	19 (41)
Kidney/pelvis	27 (59)
Therapeutic procedure	
No definitive treatment	22 (48)
Nephroureterectomy	17 (37)
Partial ureterectomy	2 (4)
Transurethral resection (TUR) of distal tumour ureter	1 (2)
Nephrectomy	4 (9)
T‐stage at therapeutic procedure for UTUC (*n* = 20)	
No malignancy	1 (5)
pTa	10 (50)
pT1	1 (5)
pT2	1 (5)
pT3	7 (35)
pT4	0 (0)
N‐stage at therapeutic procedure for UTUC (*n* = 20)	
N0	13 (65)
N1	3 (15)
Not applicable/not available	4 (20)
Mean tumour size (in mm) at pathology/radiology report (*n* = 18 available)	42 (SD: 21)
2004 WHO Grade for UTUC (*n* = 20)	
Low grade	7 (35)
High grade	11 (55)
Not applicable/not available	2 (10)

*Note*: Means and standard deviation for normally distributed continuous variables and counts and percentages for ordinal variables.

**FIGURE 1:**
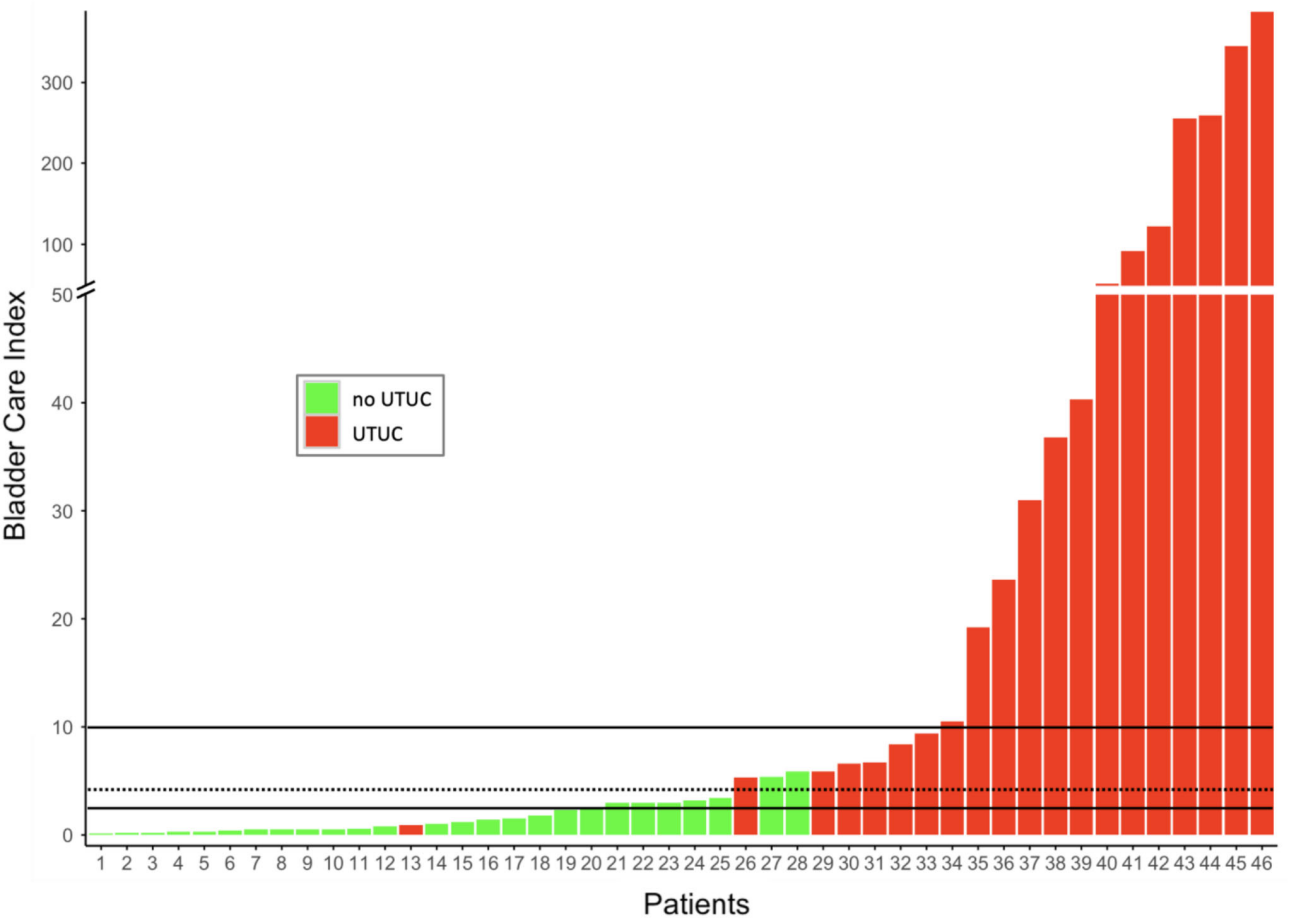
Bar chart illustrating Bladder CARE Index values in relation to UTUC status across patients. Red bars indicate UTUC‐positive cases, while green bars represent UTUC‐negative cases (benign or other cancer like renal cell carcinoma). Horizontal black lines denote manufacturer‐recommended cut‐off values: low positive (>2.5) and high positive (>10). The dotted horizontal line indicates an exploratory cut‐off value of 4.35. UTUC: upper tract urothelial carcinoma.

Using the manufacturer's cut‐off (BCI > 2.5), the test demonstrated 95% sensitivity (95% CI: 75%–100%), 69% specificity (95% CI: 48%–86%), 70% PPV (95% CI: 50%–86%) and 95% NPV (95% CI: 74%–100%) (Table 2). One of 20 patients with confirmed UTUC was false negative. This single false‐negative case (BCI 0.9) had a 12 mm pTa low‐grade tumour on nephroureterectomy following incomplete previous laser surgery (Figure 1). Using the higher cut‐off (BCI > 10), 7 of 20 patients with confirmed UTUC were false negative. With a >10 BCI cut‐off, the test demonstrated 65% sensitivity (95% CI: 41%–85%), 100% specificity (95% CI: 87%–100%), 100% PPV (95% CI: 75%–100%) and 79% NPV (95% CI: 61%–91%) (Table 2).

**TABLE 2:** Diagnostic performance metrics of different Bladder CARE Index cut‐offs and urine cytology.

Metric	BCI cut‐off >2.5		BCI cut‐off >4.35		BCI cut‐off >10		Cytology^a^	
	% (*n*/*N*)	95% CI	% (*n*/*N*)	95% CI	% (*n*/*N*)	95% CI	% (*n*/*N*)	95% CI
Sensitivity	95% (19/20)	75%–100%	95% (19/20)	75%–100%	65% (13/20)	41%–85%	11% (2/19)	1%–33%
Specificity	69% (18/26)	48%–86%	92% (24/26)	82%–100%	100% (26/26)	87%–100%	100% (22/22)	85%–100%
PPV	70% (19/27)	50%–86%	90% (19/21)	70%–99%	100% (13/13)	75%–100%	100% (2/2)	16%–100%
NPV	95% (18/19)	74%–100%	96% (24/25)	80%–100%	79% (26/33)	61%–91%	56% (22/39)	40%–72%
Accuracy	80% (37/46)	66%–91%	93% (43/46)	82%–99%	85% (39/46)	71%–94%	59% (24/41)	42%–71%

Abbreviations: BCI, Bladder CARE Index; CI, confidence interval; NPV, negative predictive value; PPV, positive predictive value.

^a^ Cytology was available in 41 patients.

**FIGURE 2:**
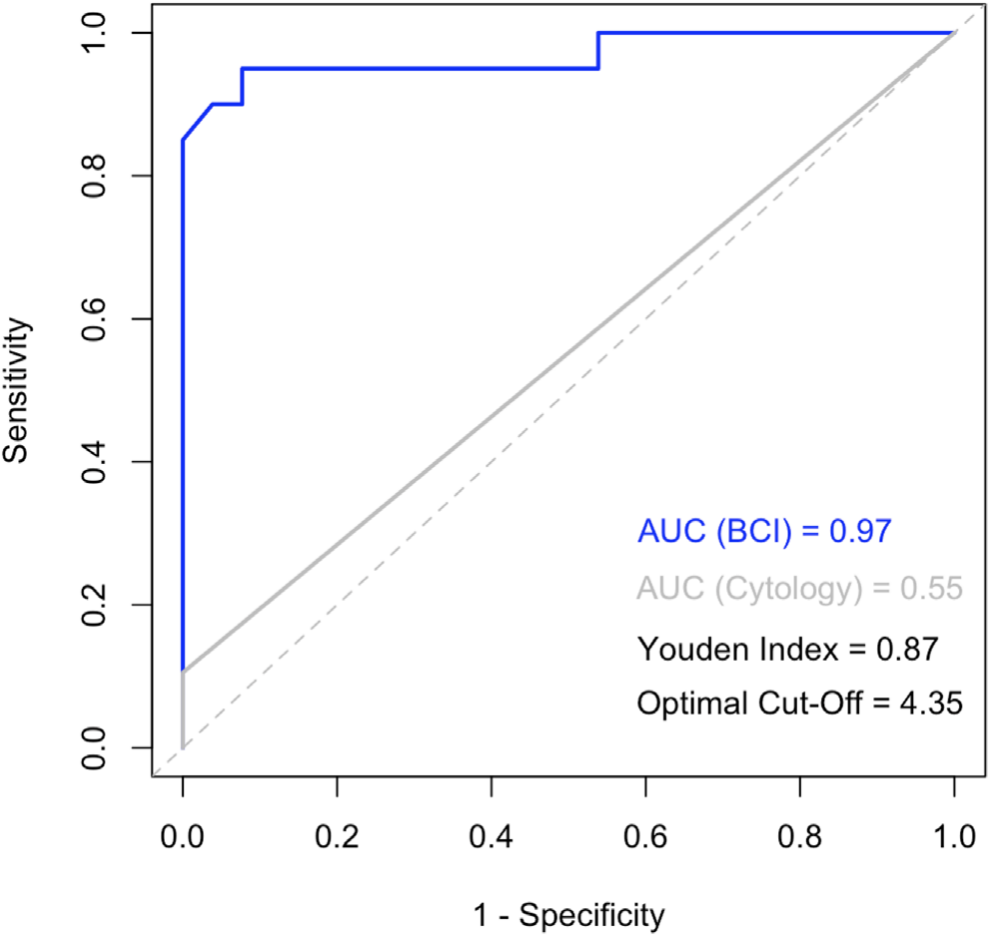
ROC curves for Bladder CARE Index (blue) and cytology (grey). The AUC of 0.97 for BCI demonstrates excellent discriminatory power between positive and negative cases. For urine cytology, the AUC of 0.55 indicates that the test is only slightly better than random chance at distinguishing between positive and negative cases the Youden Index of 0.87 suggests optimal test performance at the cut‐off point of 4.35 for BCI. The ROC curves represent sensitivity versus 1‐specificity, while the dashed diagonal line indicates random chance (AUC = 0.5). AUC: area under the curve, BCI: Bladder CARE Index, ROC: receiver operating characteristic.

The exploratory Youden Index analysis yielded an optimal cut‐off value of 4.35 (AUC 0.97, 95% CI: 0.94–1.00) (Figure 2). With this optimised cut‐off, sensitivity improved to 95% (95% CI: 75%–100%), specificity of 92% (95% CI: 82%–100%), with PPV of 90% (95% CI: 70%–99%) and NPV of 96% (95% CI: 80%–100%). In comparison, urine cytology demonstrated 11% sensitivity (95% CI: 1%–33%), 100% specificity (95% CI: 85%–100%), 100% PPV (95% CI: 16%–100%) and 56% NPV (95% CI: 40%–72%) (Table 2).

**FIGURE 3:**
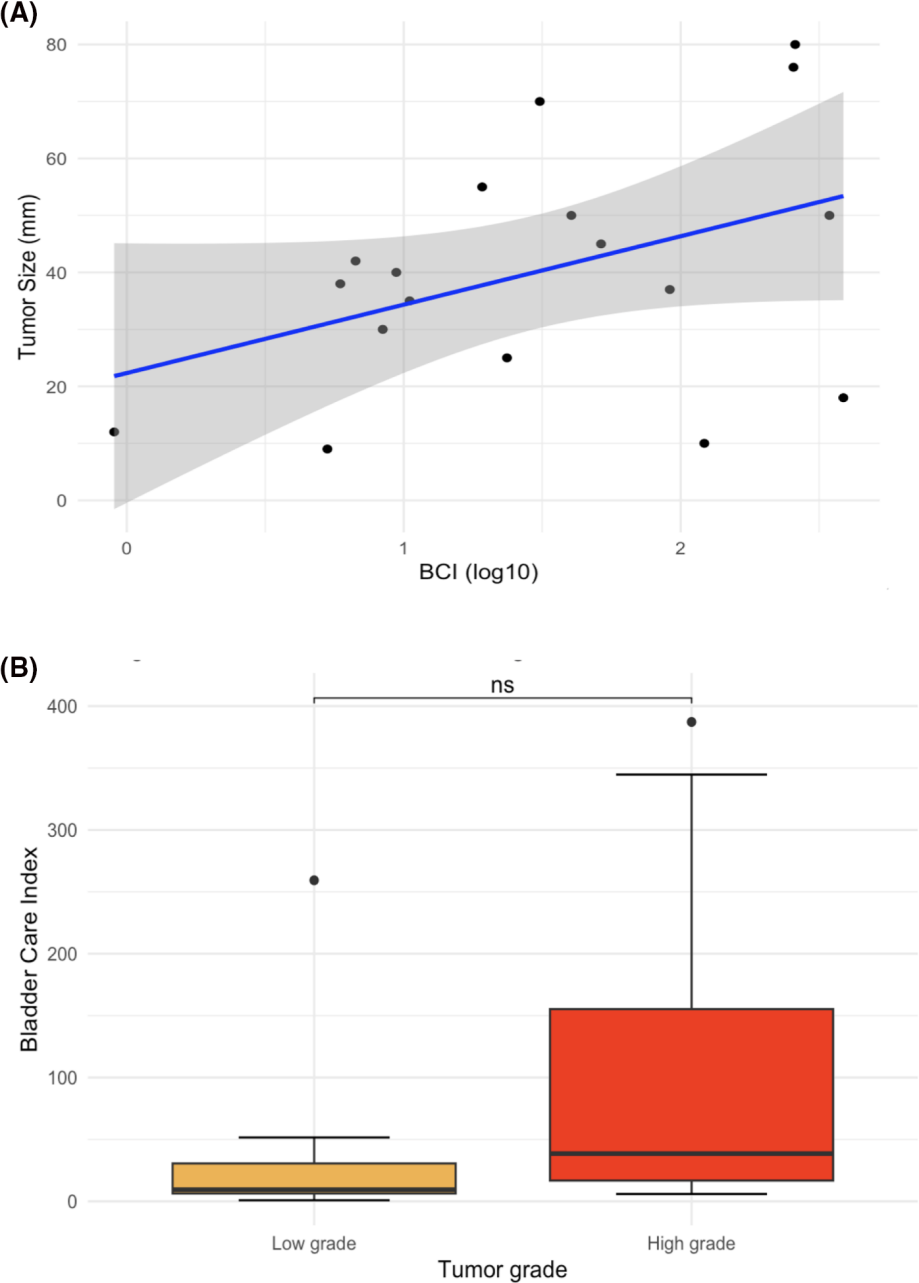
Relationship between BCI and tumour size and grade. (A) Relationship between the logarithmically transformed BCI (log10 BCI) and tumour size. Black dots represent individual patient observations. The blue line shows the fitted linear regression with 95% confidence interval (grey area), indicating a non‐significant positive association between log10 BCI and tumour size (β = 12, *p* = 0.08). (B) Distribution of BCI values according to tumour grade. Median BCI values were 9.45 (IQR 6.35–30.6), in low‐grade tumours and 38.6 (IQR 16.8–155.0) in high‐grade tumours. The difference did not reach statistical significance (Wilcoxon test, *p* = 0.14). BCI: Bladder CARE Index, log: logarithmically, ns: non‐significant, mm: millimetre.

Linear regression analysis of log10‐transformed BCI versus tumour size in 18 patients with confirmed UTUC and available tumour measurements revealed a positive association (β = 12 mm per log10 unit increase, [95% CI: −2 to 26, *p* = 0.08]), indicating that a tenfold increase in BCI corresponded to an average 12 mm increase in tumour size (Figure 3A). Patients with high‐grade pathology had higher median BCI values compared to low‐grade tumours (38.6 [IQR 16.8–155] vs. 9.45 [IQR 6.35–30.6], *p* = 0.14) (Figure 3B). One outlier with a BCI of 259 and only pTa low‐grade pathology had a large tumour filling the entire renal pelvis, precluding endoscopic management.

## 4 DISCUSSION

This study provides the first European validation of a urine‐based DNA methylation test for UTUC diagnosis. The high diagnostic accuracy suggests a potential role in the diagnostic pathway for the work‐up of patients with suspected UTUC according to CTU findings.

The Bladder CARE™ test demonstrated markedly superior accuracy compared to established diagnostic modalities in our cohort. Using the manufacturer's cut‐off of 2.5, the test achieved 95% sensitivity and 95% NPV, substantially outperforming urinary cytology, which demonstrated only 11% sensitivity. This cytology performance is consistent with reported ranges of 10%–70% for voided specimens^8^, though selective upper tract sampling can improve sensitivity to >70% in high‐grade disease.^9^ For clinical application, we propose a threshold dependent strategy. A BCI cut‐off of <2.5 was associated with a very low residual risk of UTUC in our cohort, though it cannot be considered an absolute rule‐out criterion. Patients with a BCI > 10 and concordant imaging findings may be considered for direct definitive treatment without diagnostic URS in selected clinical scenarios, as this cut‐off achieved 100% specificity (95% CI: 87%–100%) and 100% PPV (95% CI: 75%–100%) (rule‐in threshold). In contrast, patients with BCI values between 2.5 and 10 should still undergo diagnostic URS, as the moderate PPV of 70% (95% CI: 50%–86%) indicates a clinically relevant proportion of false‐positive results. Our exploratory Youden Index analysis identified an optimised cut‐off of 4.35 that balanced both objectives, achieving 92% specificity while maintaining 95% sensitivity. These findings suggest that the optimal cut‐off may vary depending on clinical context, whether the goal is to rule out disease, confirm disease, or balance both considerations.

We believe that novel diagnostic tests with characteristics similar to our results have the potential to define a new diagnostic pathway as we suggest in Figure S1, but this requires further prospective validation before being used clinically. Current diagnostic pathways frequently necessitate URS, which delays definitive treatment by 2–4 weeks and carries risks including bleeding (9%),^2^ ureteral strictures (7%),^2^ infections (9%)^2^ and potential tumour seeding.^3^, ^4^ Despite providing direct visualization and biopsy capabilities, URS specimens prove inconclusive in a significant portion of patients (6%–8% non‐diagnostic, 26%–32% un‐staged and 1%–12% un‐graded),^10^ further delaying treatment.

Our findings support a modified diagnostic algorithm: patients with an elevated BCI > 10 and concordant imaging suggesting UTUC could proceed directly to radical (nephro‐)ureterectomy when indicated, bypassing diagnostic URS. This approach would eliminate URS‐related complications and expedite treatment. Additionally, avoiding URS‐related tumour manipulation could improve oncological outcomes by reducing intravesical recurrence risk, as demonstrated by van Doeveren et al.^4^ Meta‐analyses have demonstrated that diagnostic URS before radical nephroureterectomy significantly increases intravesical recurrence rates (hazard ratio 1.44, 95% CI: 1.29–1.61), with intravesical recurrence occurring in up to 35%–50% of patients, though without negatively impacting oncological outcomes.^11^ Conversely, intermediate results (BCI 2.5–10) should be interpreted cautiously and confirmed by diagnostic URS to avoid overtreatment of false‐positive cases.

Beyond streamlining the decision to bypass URS, Bladder CARE™ test offers potential for non‐invasive grade determination, which could identify patients most likely to benefit from neoadjuvant chemotherapy or be eligible for kidney‐sparing approaches. Indeed, our preliminary data suggest that high‐grade tumours have higher median BCI values compared to low‐grade pathology. This diagnostic stratification aligns with the growing clinical emphasis on optimising preoperative systemic therapy. Increasing evidence demonstrates improved survival with neoadjuvant approaches^12^, ^13^, ^14^ with immuno‐chemotherapy combinations in particular demonstrating favourable safety profiles.^15^, ^16^ Ongoing clinical trials are currently investigating various regimens, including neoadjuvant chemotherapy combinations (NCT04574960, NCT02969083) and immuno‐chemotherapy approaches incorporating checkpoint inhibitors such as Durvalumab or Toripalimab (NCT04099589, NCT04628767).

Without reliable grade assessment, clinicians face a critical dilemma. Proceeding directly to surgery risks potentially undertreating high‐grade disease that would benefit from chemotherapy. On the other hand, administering neoadjuvant therapy to all patients risks overtreatment, particularly in large tumours that ultimately prove to be only pTa low‐grade with limited benefit from chemotherapy. This decision is particularly crucial as only 19% of patients remain eligible for adjuvant therapy post‐nephroureterectomy due to renal function decline.^17^ Similarly, kidney‐sparing treatments including segmental ureterectomy or endoscopic laser ablation offer valuable alternatives but carry risks of incomplete resection and require accurate grade assessment for appropriate patient selection.

Future applications extend beyond primary diagnosis. Bladder CARE™ shows promise for surveillance following kidney‐sparing treatment, monitoring contralateral kidneys and screening high‐risk populations including patients with Lynch syndrome. Serial BCI monitoring might assess treatment response or detect recurrence, though prospective validation is essential.

Several limitations warrant consideration. The retrospective design and sample size of 46 patients, while adequate for the predefined diagnostic accuracy objectives and given the rarity of UTUC (2 per 100 000 annually),^18^ limit generalizability. Furthermore, the small cohort size limits the precision of PPV estimates, particularly at lower thresholds, and reinforces the need for larger prospective trials before clinical implementation. Although prospective sample collection and standardised testing strengthen validity, the single‐centre nature and predominantly Caucasian population necessitate multicentre validation in diverse cohorts. The false‐negative case, a small low‐grade tumour following incomplete laser surgery, highlights that negative results cannot definitively exclude UTUC in all contexts, particularly after prior intervention.

## 5 CONCLUSION

This novel urine‐based DNA methylation test demonstrates diagnostic accuracy with the potential to modify UTUC management algorithms. At higher thresholds (e.g., BCI > 10), the test could allow direct progression to definitive treatment without diagnostic URS, reducing complications and expediting care. The correlation between BCI levels and tumour grade addresses critical unmet needs in both neoadjuvant therapy selection and kidney‐sparing surgery planning. While multicentre validation remains essential, this non‐invasive molecular test represents a promising advance in UTUC diagnostics with potential to improve both diagnostic efficiency and therapeutic decision‐making.

## AUTHOR CONTRIBUTIONS

Christian Daniel Fankhauser had full access to all the data in the study and takes responsibility for the integrity of the data and the accuracy of the data analysis.

*Study concept and design*: Kaufmann, Fankhauser, Chew, Piatti. *Acquisition of data*: Kaufmann, Röthlin, Fankhauser, Baumeister, Mattei. *Analysis and interpretation of data*: Kaufmann, Fankhauser, Chew, Piatti. *Drafting of the manuscript*: Kaufmann, Fankhauser. *Critical revision of the manuscript for important intellectual content*: Kaufmann, Fankhauser, Röthlin, Chew, Piatti, Baumeister, Mattei. *Statistical analysis*: Kaufmann, Fankhauser, Chew. *Obtaining funding*: None. *Administrative, technical or material support*: Kaufmann, Fankhauser, Chew, Piatti. *Supervision*: Kaufmann, Fankhauser. *Other*: None.

## CONFLICT OF INTEREST STATEMENT

P. Piatti and Y.C. Chew are employees of Pangea Laboratory and Zymo Research Corp, which provided the Bladder CARE™ test kits. The remaining authors have no conflicts of interest to declare.

## Supporting information


**Figure S1** Proposal for a theoretical and hypothesis‐generating clinical pathway incorporating the Bladder Care Test in the decision‐making process of patients with suspected UTUC. “BCI very high” indicates extremely elevated values that may identify patients eligible for neoadjuvant therapy. However, the exact threshold for “very high” BCI still needs to be defined in future studies, particularly with regard to reliably discriminating between low‐ and high‐grade disease before neoadjuvant therapy can be recommended. Abbreviations: BCI: Bladder Care Index, CT: computerized tomography, URS: Ureterorenoscopy, UTUC: Upper tract urothelial carcinoma.
