# Supplementary material for: Validation of the diagnostic accuracy of a urine‐based DNA methylation marker test in patients with upper urinary tract lesions
Source: BJUI Compass. 2026 Mar 19;7(3):e70195. doi: 10.1002/bco2.70195 (PMC13093633; doi:10.1002/bco2.70195)

**Supplementary Figure 1** Proposal for a theoretical and hypothesis-generating clinical pathway incorporating the Bladder Care Test in the decision-making process of patients with suspected UTUC. “BCI very high” indicates extremely elevated values that may identify patients eligible for neoadjuvant therapy. However, the exact threshold for “very high” BCI still needs to be defined in future studies, particularly with regard to reliably discriminating between low- and high-grade disease before neoadjuvant therapy can be recommended. Abbreviations: BCI: Bladder Care Index, CT: computerized tomography, URS: Ureterorenoscopy, UTUC: Upper tract urothelial carcinoma


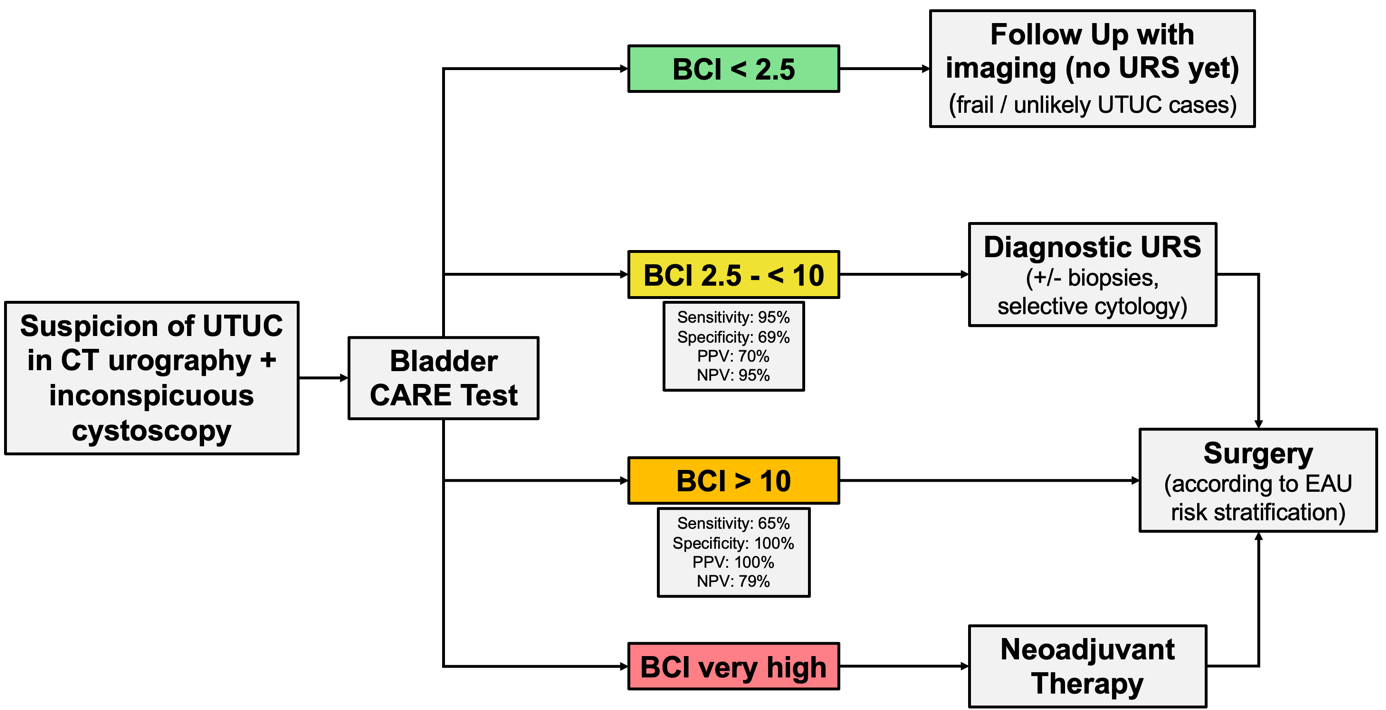

Supplement: Supplementary file 1 — Figure S1 Proposal for a theoretical and hypothesis‐generating clinical pathway incorporating the Bladder Care Test in the decision‐making process of patients with suspected UTUC. “BCI very high” indicates extremely elevated values that may identify patients eligible for neoadjuvant therapy. However, the exact threshold for “very high” BCI still needs to be defined in future studies, particularly with regard to reliably discriminating between low‐ and high‐grade disease before neoadjuvant therapy can be recommended. Abbreviations: BCI: Bladder Care Index, CT: computerized tomography, URS: Ureterorenoscopy, UTUC: Upper tract urothelial carcinoma. [file BCO2-7-e70195-s001.docx]
